# Test–Retest Variability and Reliability of Volitional and Nonvolitional Assessments of Muscle Endurance in Healthy Humans

**DOI:** 10.1249/MSS.0000000000003866

**Published:** 2025-10-15

**Authors:** ALAN J. METCALFE, EDWARD MILLS, TRUDIE CHALDER, CAROLINE J. JOLLEY, GERRARD F. RAFFERTY

**Affiliations:** 1 Centre for Human and Applied Physiological Sciences (CHAPS), Faculty of Life Sciences and Medicine, Denmark Hill Campus, King’s College London, London, UNITED KINGDOM; 2 School of Human and Social Sciences, University of West London, Brentford, London, UNITED KINGDOM; 3 Department of Psychological Medicine, Institute of Psychiatry, Psychology and Neuroscience, King’s College, London, UNITED KINGDOM; 4 Department of Respiratory Medicine, King’s College Hospital NHS Foundation Trust, London, UNITED KINGDOM

**Keywords:** EFFORT INDEPENDENT, ELECTRICAL STIMULATION, HAND GRIP, QUADRICEPS, STRENGTH

## Abstract

**Purpose:**

Muscle endurance is commonly assessed using repeated or sustained volitional contractions, but measurement can be affected by subject cooperation and motivation. Nonvolitional techniques employing direct muscle stimulation may overcome the shortcomings associated with volitional techniques. We have therefore examined the variability and reliability of both volitional and nonvolitional techniques to assess quadriceps and handgrip endurance.

**Methods:**

Fourteen healthy participants (10 male, age 30 ± 7 yr, height 173.3 ± 9.2 cm, weight 67.0 ± 12 kg) completed measurements of strength and endurance on three occasions. Hand grip and quadriceps strength were determined from hand grip maximal voluntary contraction (HGMVC) and quadriceps maximal voluntary contraction (QMVC), respectively. Volitional hand grip endurance was determined as the time to task failure during a sustained contraction at 50% HGMVC. Volitional quadriceps endurance was assessed using repeated 5-s isometric contractions at 60% QMVC with 3 s rest until task failure. Nonvolitional quadriceps endurance was assessed using repetitive transcutaneous electrical stimulation (30 Hz, 250 ms on, 750 ms off for 180 s) at 30% QMVC. Endurance was taken as the time for the force to fall to 70% of the initial force. Variability was determined using the coefficient of variation, and reliability using intraclass correlation coefficient.

**Results:**

Volitional hand grip endurance demonstrated fair variability (20.5%) and good reliability (0.81). Volitional quadriceps endurance demonstrated good variability (15.2%) and good reliability (0.76) while nonvolitional quadriceps endurance demonstrated good variability (16.4%) and excellent reliability (0.92). Overall, HGMVC and QMVC demonstrated either good or very good variability and either good or excellent reliability.

**Conclusions:**

Both volitional and nonvolitional measures of endurance in the hand and quadriceps muscles are reliable. Nonvolitional measures of muscle endurance may be helpful in cohorts unable to perform volitional maneuvers.

Measuring skeletal muscle strength and endurance is a fundamental assessment employed in research studies in healthy participants and patient populations (1–3). Reliable measures are critical in clinical populations to optimize exercise prescription, improve rehabilitation outcomes, and manage neurological conditions. Muscle fatigue is a physiological process defined as a loss of force resulting from sustained muscular activity, which is reversible by rest (4), and the capacity to resist this loss of force over time induced by fatigue is termed muscle endurance (5). Assessment of muscle endurance commonly relies on volitional maneuvers such as sustained submaximal contractions (3,6–8) or repeated maximal intermittent contractions (9,10). Volitional techniques, by their nature, require participant cooperation and motivation, which can both limit their application and negatively influence test performance and variability. The use of nonvolitional tests to accurately assess muscle endurance in which the element of motivation is removed could be of significant practical benefit in clinical populations by guiding rehabilitation to maintain or improve endurance in individuals with reduced exercise capacity, such as in chronic cardiovascular or respiratory disease, spinal cord injury (SCI), neuromuscular disorders, or post critical care. Preserving muscle endurance in these conditions may also slow or prevent secondary complications such as osteoporosis, metabolic disease, muscle atrophy, and a shift toward a glycolytic muscle phenotype.

Despite the frequent use of techniques to assess endurance, there are few data concerning their variability and reliability. Zech et al. (11) showed high test–retest reliability for quadriceps endurance using a sustained contraction at 50% of maximum voluntary contraction (MVC) force, but indicated that high motivation of subjects and strong verbal encouragement were essential to reach task failure. Frykholm et al. (12) investigated between-day test–retest feasibility and reliability of isokinetic, isometric, and isotonic quadriceps endurance in chronic obstructive pulmonary disease (COPD) patients. While tests of quadriceps endurance were feasible, their reliability varied from low to very high. Similar findings have been reported for hand grip endurance. Low to high reliability has been reported when using intermittent maximal contractions or sustained protocols, submaximal contraction at 50% MVC (13–16).

We and others (8,17) have previously applied nonvolitional techniques, such as repetitive transcutaneous electrical (17) and magnetic stimulation (8), to assess muscle endurance. Such an approach removes the volitional component of testing by directly stimulating the muscle and therefore minimizes the influence of participant motivation and potentially improves test reliability. Such nonvolitional techniques also allow assessment of endurance to be performed in populations unable to adequately perform volitional maneuvers.

The aim of this study was, therefore, to examine the test–retest variability and reliability of techniques to assess muscle endurance in the upper and lower limbs in healthy participants and to compare test–retest variability and reliability of volitional and nonvolitional techniques to assess muscle endurance in the lower limb. We also measured the variability and reliability of volitional measures of strength and voluntary activation (VA) for comparative purposes. We hypothesized that measurements of muscle endurance would be reliable between days and that nonvolitional techniques would be more reliable and with lower variability than volitional approaches. While measurements of strength and endurance are fundamental measurements applied in research and clinical assessment, there are few data examining the relationship between these two variables, and the results are inconsistent. In addition, while hand grip strength has been used as an estimate of whole body strength, adopting a similar approach for measuring muscle endurance may not be appropriate. A better understanding, therefore, of the relationship between hand grip and quadriceps muscle endurance would be of benefit. A secondary aim of the study was, therefore, to examine the relationships between muscle strength and endurance and the relationships between measures of endurance performed in the upper and lower limbs.

## MATERIALS AND METHODS

### Participants

Handgrip and quadriceps strength and endurance were measured on three occasions in 14 healthy participants (age 30 ± 7 yr, height 173.3 ± 9.2 cm, weight 67.0 ± 12 kg, body mass index 22.4 ± 3.3, sex [male:female 10:4]). Healthy participants with no history of cardiorespiratory or neuromuscular disease were recruited for this study. Ethical approval was obtained from King’s College London Ethics Committee (study ref HR/DP-21/22-26429), and written informed consent was obtained from all participants before any testing commenced.

### Study protocol

For each study visit, participants completed five measurements of strength and endurance in the same order on each occasion. Measurements of maximal quadriceps strength for both dominant and nondominant limbs were performed first, followed by hand grip strength for both dominant and nondominant limbs. The nonvolitional test of muscle endurance was then performed on the dominant leg, followed by the volitional test of endurance on the nondominant leg. Testing was completed with the measurement of hand grip endurance in the dominant limb. All measurements of strength and endurance were performed on three separate occasions, with 2–4 d between each testing session. Participants were asked to refrain from strenuous exercise in the days between each testing session, and testing was performed under the same environmental conditions and, when possible, at the same time of day. The intake of ergogenic substances before testing, such as caffeine, was not controlled. Participants received full instruction on how to perform each test by an experienced investigator and were observed closely to ensure the correct technique was followed. Participant anthropometrics were recorded on the first visit to the laboratory, with height measured using a stadiometer (Seca GmbH, Hamburg, Germany) and weight using calibrated scales (Seca GmbH). Limb dominance was determined by participant self-report. Hand grip and quadriceps strength measured on each testing occasion were used to set the endurance protocols at the appropriate intensity.

### Quadriceps strength (quadriceps maximal voluntary contraction)

Quadriceps strength was assessed by sustained (3–5 s) maximal isometric voluntary contractions. Participants were seated upright on a custom-designed testing chair with the back set at 90º and with the knees flexed at 90º. Quadriceps force was measured in both dominant (quadriceps maximal voluntary contraction [QMVC]-D) and nondominant (QMVC-ND) legs at the ankle using an inextensible strap positioned 2 cm above the lateral malleolus connected to a force transducer (Strainstall, Cowes, United Kingdom). Force signals were amplified, recorded, and displayed in real time using LabChart software (version 8, ADInstruments, Oxford, UK), with an analog-to-digital conversion rate of 100 Hz (Powerlab 16, ADInstruments, Oxford, United Kingdom). The maximum 1-s average over the plateau in quadriceps force was used to measure QMVC. No warm-up was performed, but at least five maximal efforts were performed for both dominant and nondominant legs with at least 30 s between efforts. Additional efforts were performed if the operator considered the first five efforts to be submaximal. This decision was based on whether the fifth measurement was the largest of the testing session or if the second largest measurement was less than 90% of the largest. Loud verbal encouragement was given during each effort. The largest value achieved across all efforts performed was reported as the QMVC-D and QMVC-ND.

### Voluntary activation (VA)

To determine the level of muscle recruitment and assess the participant's effort during QMVC, the twitch interpolation technique (1,18) was performed in the dominant leg to assess VA. Two carbon rubber stimulating electrodes (130 mm × 100 mm) were placed over the quadriceps femoris of the participant’s dominant leg. The two stimulating electrodes were placed transversely on the frontal surface of the thigh with the anode positioned approximately 3 cm above the upper border of the patella across the heads of the quadriceps muscle. The cathode was placed proximal to the anode with at least a 3-cm gap from the edge of the anode and covering the motor point. Quadriceps muscle stimulation was performed using 300 μs square wave pulses delivered from a constant current stimulator (DS7AH, Digitimer, Welwyn Garden City, United Kingdom). The stimulus intensity used to determine VA was that used when assessing quadriceps nonvolitional endurance, that is, to elicit a quadriceps force equivalent to 30% of QMVC. The assessment of VA was performed during the measurement of QMVC-D with an interpolated twitch delivered during the QMVC maneuver and a potentiated twitch delivered approximately 2 s after the MVC maneuver. VA was determined as (1 − interpolated Tw/potentiated Tw) × 100.

### Hand grip strength (hand grip maximal voluntary contraction)

A hydraulic hand dynamometer (Baseline, Fabrication Enterprises, Inc., White Plains, NY) was used to measure hand grip strength using the standard procedure. The participant was seated, shoulder adducted, and elbow flexed at 90° with the forearm and wrist in a neutral position. The dynamometer was placed in the participant's hand, and they were instructed to squeeze as hard as possible, applying grip force smoothly, without any rapid jerking motion. Loud verbal encouragement was given during each effort. No warm-up was performed, but at least five sustained (3–5 s) maximal efforts were performed for both dominant (hand grip maximal voluntary contraction [HGMVC]-D) and nondominant (HGMVC-ND) hands with at least 30 s between efforts. Additional efforts were performed if the operator considered the first five efforts to be submaximal. This decision was based on whether the fifth measurement was the largest of the testing session or if the second largest measurement was less than 90% of the largest. The largest value achieved across all efforts performed was reported as the HGMVC-D and HGMVC-ND.

### Quadriceps volitional endurance—nondominant leg

A Tabata-style protocol was used to assess volitional quadriceps endurance (QEvol) in the nondominant leg, which involved participants performing repeated 5-s isometric quadriceps contractions at 60% of QMVC followed by 3 s rest until task failure. Task failure was defined as three consecutive contractions below 60% QMVC, with task completion defined as completing 75 separate contraction/relaxation cycles (600 s). Visual feedback on force production and target level was provided from the displayed trace on the data acquisition system. A preset interval timer was used to track cycle number and contraction/relaxation periods. Verbal encouragement was given throughout the test.

### Quadriceps nonvolitional endurance—dominant leg

Nonvolitional muscle endurance was assessed in the dominant leg using repetitive transcutaneous electrical stimulation (rTES) with cyclical trains of 30 Hz, 300 μs square wave stimulations delivered by a constant current stimulator (DS7AH, Digitimer), administered with a duty cycle of 250 ms on and 750 ms off for 180 s. Electrical stimulation was delivered using the carbon rubber stimulating electrodes placed over the quadriceps femoris to assess VA. The intensity of stimulation was set to elicit an initial quadriceps force equivalent to 30% of QMVC. Quadriceps force production was recorded continuously by the acquisition system, with an analog-to-digital sampling at 100 Hz (Powerlab 16, ADInstruments). Subjects were instructed to relax fully during testing. Only assessments in which quadriceps force returned to baseline during the off phase of stimulation were included in the analysis. Quadriceps nonvolitional endurance was calculated as the time for the quadriceps force to fall to 70% (QET70) of the maximum force produced during the 180 s.

### Hand grip endurance

A digital hand grip force transducer (MLT003/D Grip Force Transducer, ADInstruments, Dunedin, New Zealand) was used to assess volitional hand grip endurance in the dominant hand (HGEvol). Use of the digital handgrip force transducer allowed force to be recorded and displayed continuously by the acquisition system (LabChart) throughout the endurance protocol. The digital hand grip force transducer was calibrated with a known weight before each testing session, and agreement with the hydraulic dynamometer was confirmed. Participants were positioned in the same way as that used for HGMVC measurement, seated, shoulder adducted, and elbow flexed at 90° with the forearm and wrist in a neutral position. Participants were asked to target a sustained handgrip contraction at 50% of HGMVC to task failure. Due to the inherent variability in force commonly seen during sustained volitional contractions, HGEvol was defined as the time at which force fell below 40% of HGMVC. Pilot testing indicated that force fell rapidly at task failure, and using this approach therefore provided a clear time point for task failure. Visual feedback on force production and target level was provided using the displayed trace on the data acquisition system. Loud verbal encouragement was given throughout the test.

### Statistics

Data are expressed as means ± standard deviation (SD). Reliability was assessed as the intraclass correlation coefficient (ICC) using a two-way random effects model with a single measure (Atkinson and Nevill (19)). The ICC values for poor, moderate, good, and excellent reliability are <0.5, 0.5–0.75, 0.75–0.9, and >0.90, respectively (20). Variability was assessed using the coefficient of variation (CV) determined as follows: CV = SD/mean × 100. The CV values for very good, good, fair, and poor variability are <10%, 10%–20%, 20%–30%, and >30%, respectively (21). The ICC calculations were performed using SPSS version 29 (IBM Corp, Armonk, NY). The CVs were calculated using Microsoft Excel (Microsoft Corporation, Redmond, WA). The association between measurements was assessed using Spearman rank correlation coefficient (*R*^2^) using StatNow version 19.5 (StataCorp LLC, College Station, TX) with a *P* value <0.05 taken to be significant. The strength of correlation coefficients was interpreted using Evans classification (22); *r* = 0–0.19: very weak, 0.2–0.39: weak, 0.40–0.59: moderate, 0.6–0.79: strong, and 0.8–1: very strong. The mean value for each of the strength and endurance measures across the three testing sessions was used in the calculation. All figures were created using GraphPad Prism software (version 10.0.0, GraphPad Software, Boston, MA).

## RESULTS

**FIGURE 1:**
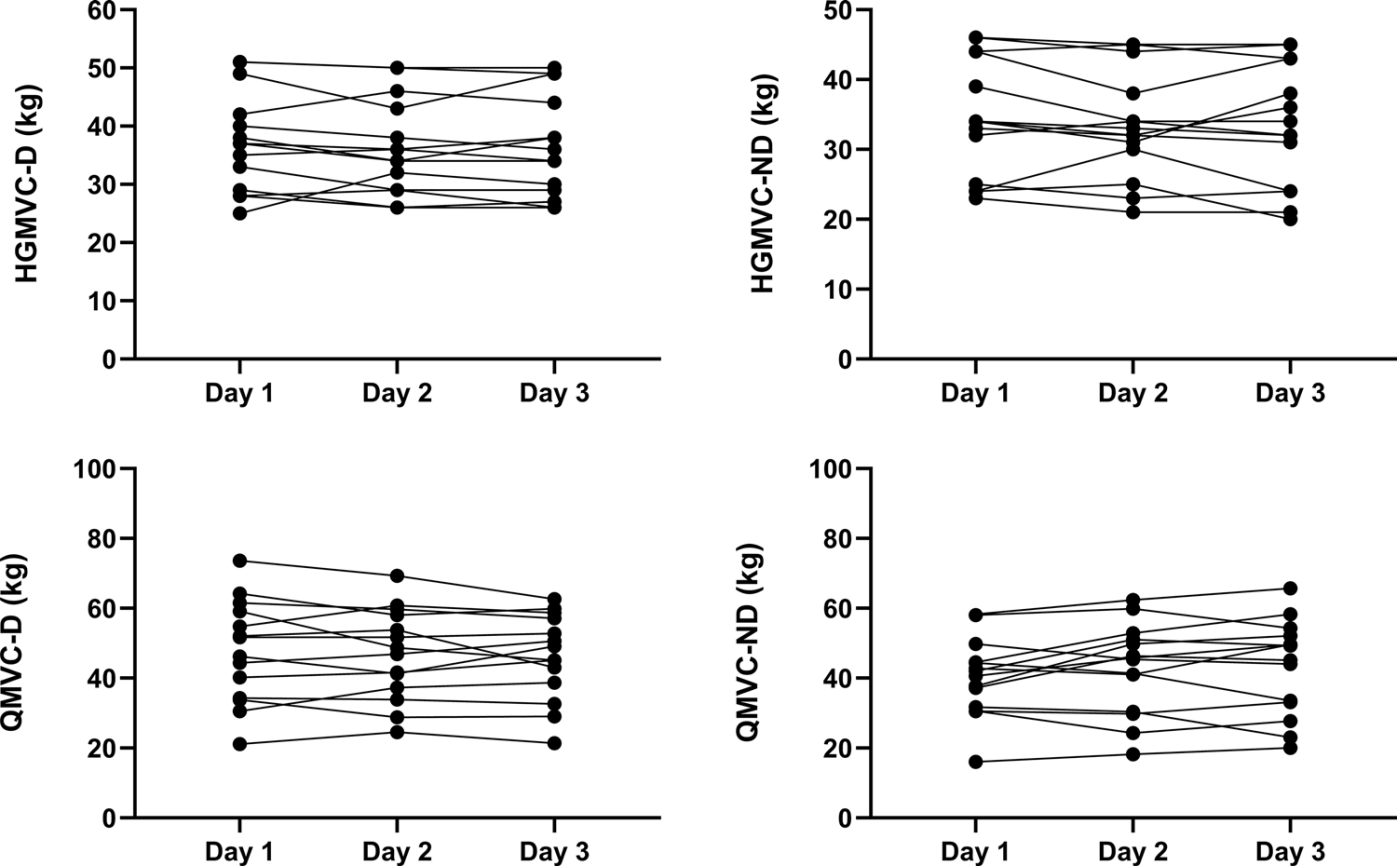
Individual values of dominant (D) and nondominant (ND) hand grip (HGMVC) and quadriceps (QMVC) strength across the 3 d of testing (*N* = 14).

**FIGURE 2:**
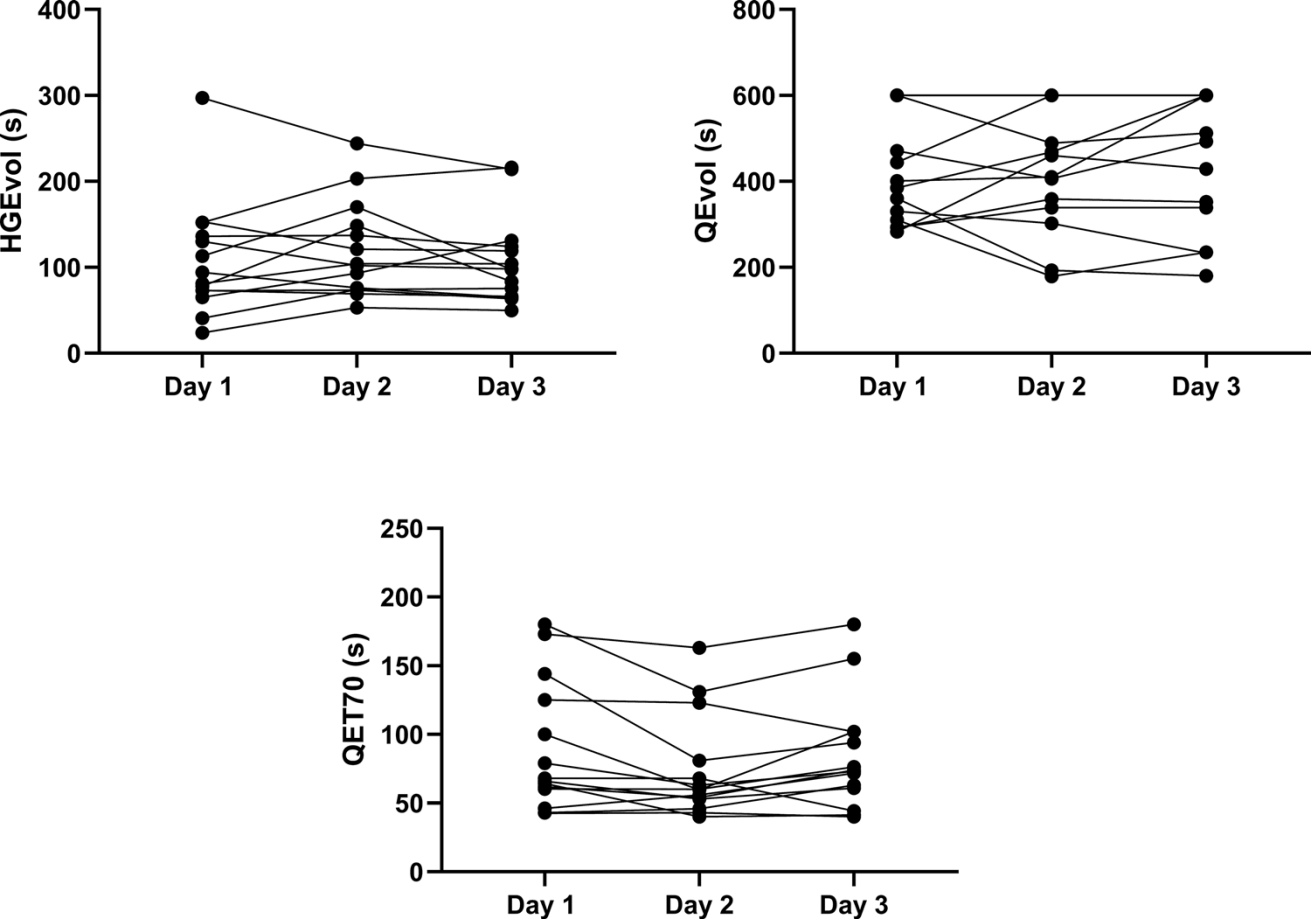
Individual values for volitional hand grip (HGEvol) and volitional quadriceps (QEvol) endurance and nonvolitional quadriceps endurance (QET70) across the 3 d of testing (*N* = 14).

Measurements of handgrip and quadriceps strength (Fig. 1) and endurance (Fig. 2) were completed on three occasions in all 14 healthy subjects. The group mean value and within occasion CV (%) for the measurements of strength, along with the variability and reliability for both strength and endurance measurements across the 3 testing sessions, are shown in Table 1.

**TABLE 1.** Within and between occasion measurements of strength and endurance.

	Within Occasion			Between Occasion		
	Day 1, Mean ± SD	Day 2, Mean ± SD	Day 3, Mean ± SD	Mean ± SD	Coefficient of Variation (%)	Intraclass Correlation Coefficient (95% Confidence Interval)
HGMVC-D, kg CV, %	37.4 ± 8.6 5.8%	36.4 ± 8.2 4.9%	36.4 ± 8.7 5.4%	36.7 ± 8.3	5.2	0.94 (0.87–0.98)
HGMVC-ND, kg CV, %	34.4 ± 8.4 5.8%	33.4 ± 7.6 6.2%	33.4 ± 8.8 6.8%	33.7 ± 8.1	6.1	0.92 (0.83 -0.97)
QMVC-D, kg CV, %	47.7 ± 14.7 9.9%	46.9 ± 13 8.0%	46.1 ± 12.3 7.2%	46.9 ± 13.0	7.4	0.91 (0.80–0.96)
QMVC-ND, kg CV, %	40.3 ± 11.2 6.5%	32.8 ± 13 6.5%	43.2 ± 13.7 7.0%	42.1 ± 12.1	10.6	0.87 (0.72–0.95)
HGEvol, s	107.8 ± 67.2	119.1 ± 55.8	107.6 ± 51.7	115.5 ± 54.9	20.5	0.81 (0.61–0.93)
QEvol, s	426.4 ± 126.8	428.9 ± 144.5	455.2 ± 159.1	436.8 ± 123.5	15.2	0.76 (0.53–0.91)
QET70, s	89.5 ± 53.5	74.4 ± 45.4	84.7 ± 48.2	82.7 ± 40.4	17.2	0.96 (0.89–0.98)

Within occasion measurements show mean ± SD and mean within occasion CV for measures of strength and mean ± SD measures of endurance on each day of testing. Between occasion values are mean ± SD variability (CV [%]) and reliability (intraclass correlation coefficient [95% confidence interval]) of strength and endurance measurements across the three visits. All *N* = 14.

### Strength

HGMVC-D, HGMVC-ND, and QMVC-D demonstrated very good variability (5.2%, 6.1%, and 7.4%, respectively) and excellent reliability (0.94, 0.92, and 0.91, respectively). QMVC-ND demonstrated good variability (10.6%) and reliability (0.87). The mean (SD) measurements of VA over the 3 d of testing were 77.7% (11.5), 75.8% (12.8), and 77.1% (10.2) with very good variability (CV: 9.1%), but poor reliability (ICC [95% CI] 0.48 [0.12–0.78]).

### Endurance

HGEvol demonstrated fair variability (20.5%) and good reliability (0.81) while QEvol demonstrated good variability (15.2%) and good reliability (0.76). QET70 demonstrated good variability (17.2%) and excellent reliability (0.96).

### Correlations between strength and endurance

Strong, statistically significant, correlations were observed between HGMVC-D and HGMVC-ND (*R*^2^ = 0.90, *P* < 0.0001) and between QMVC-D and QMVC-ND (*R*^2^ = 0.88, *P* < 0.0001), as well as between QMVC-D and HGMVC-D (*R*^2^ = 0.88, *P* < 0.0001) and between QMVC-ND and HGMVC-ND (*R*^2^ = 0.88, *P* < 0.0001).

No significant relationships (*r* = very weak or weak) were observed between muscle strength and volitional measures of endurance in the hand (HGEvol vs HGMVC-D, *r* = −0.048) or quadriceps (QEvol vs QMVC-ND, *r* = −0.088) or between volitional endurance in the hand and quadriceps (HGEvol vs QEvol, *r* = 0.38), and between strength and nonvolitional endurance in the quadriceps (QMVC-D vs QEvol, *r* = −0.057). There was also no significant relationship observed between volitional and nonvolitional quadriceps endurance (QEvol vs QET70, *R*^2^ = 0.28, *P* = 0.054).

## DISCUSSION

The primary purpose of this study was to examine the variability and reliability of volitional and nonvolitional techniques to assess muscle endurance in both the hand and quadriceps muscles. We also measured the variability and reliability of volitional measures of strength for comparative purposes and examined the relationship between measures of strength and endurance.

Volitional hand grip endurance showed fair variability (20.5%) and good reliability (0.81) while quadriceps volitional endurance showed similar levels of variability (good 15.2%) and reliability (good 0.76). Nonvolitional measurement of quadriceps endurance exhibited good variability (17.2%) and excellent reliability (0.96). The excellent test–retest reliability of nonvolitional measurement of muscle endurance indicates that muscle fatigability can be consistently measured and potentially provides a robust metric for assessing muscular endurance in participants who are unable to perform volitional maneuvers.

The findings of the current study are similar to previous research, indicating that both volitional measures of hand grip and quadriceps strength and endurance are, in general, reliable measures for assessing muscle function in both healthy (11,23) and clinical populations (12,24). While volitional handgrip endurance demonstrated fair variability but good reliability, volitional measures of quadriceps endurance showed both good variability and reliability. The current study was undertaken in healthy participants, and the results may not, therefore, be generalizable to naive patient populations in which accuracy of testing may be affected by participant cooperation or motivation due to anxiety, breathlessness, or pain. Previous studies have highlighted the problem with participant motivation (25), although our results suggest that a submaximal 600-s Tabata-style protocol to measure volitional quads endurance could be used reliably in healthy subjects to examine muscle endurance. Regardless of whether sustained or intermittent protocols are used to assess endurance, assessing participant perception during volitional performance (i.e., rating of perceived exertion, pain) would provide some insight into such measures in future work.

While our data support the use of volitional measures of endurance, volitional testing cannot be applied reliably in populations of patients unable to comply with commands such as those in critical care. A reliable test that is independent of subject cooperation and motivation allows measures of endurance to be applied in cohorts of patients that traditionally cannot be assessed using a volitional approach. In addition, applying both nonvolitional and volitional tests of quadriceps muscle endurance may allow underlying mechanisms to be elucidated. Reductions in volitional endurance with no change in nonvolitional endurance measures may suggest that task performance is limited by central mechanisms associated with alterations within the brain and central nervous system rather than any change in peripheral muscle function.

The volitional quadriceps endurance was a Tabata-style protocol in which three participants completed the entire protocol (600 s). We further examined these data to determine whether successfully completing the test would exert a ceiling effect on the test and introduce bias to the results. Removing the three participants who completed the test showed no difference in the level of variability (392 ± 112 vs 436 ± 132 s). Quadriceps volitional and nonvolitional endurance were assessed using repeated submaximal contractions, whereas hand grip endurance testing employed a sustained contraction. While assessments of handgrip endurance using repeated maximal contractions have been performed previously, studies have reported low to high reliability (13–15) when using this approach. We wished to use a repeated submaximal test in the current study to avoid issues associated with participant motivation and blood flow occlusion, and pilot testing in the current study had shown that subjects were easily able to complete a 10-min intermittent protocol, which may have imposed a ceiling effect on the test.

We used rTES to measure nonvolitional quadriceps endurance. Transcutaneous stimulation will only recruit a portion of the quadriceps muscle, and the quadriceps force produced was submaximal (30% of MVC). It is also not possible to position the pads so that exactly the same portion of muscle is stimulated on all occasions (26). Transcutaneous electrical stimulation can also be painful due to stimulation of skin nociceptors and the strength of the induced contraction, which is related to the stimulating currents, electrode size, and position (27) (Gravholt #13674) (28) . Although not measured directly, rTES was well tolerated by the participants in the current study, which we attributed to the use of an intermittent stimulation protocol, large stimulating electrodes, and relatively low induced force (30% QMVC) and duration of the protocol (3 min). Despite such limitations, the variability and reliability of nonvolitional quadriceps endurance were good and excellent, respectively, and correlated with volitional measurement of quadriceps muscle endurance.

Variability of measures of strength was included for comparative purposes. Both HGMVC and QMVC strength exercises were within or above the normal range (29) and demonstrated at least good variability and reliability (Table 1). Hand grip and quadriceps MVC are widely used to assess muscle strength and age-associated decline in both healthy and clinical populations (24,30). It is, therefore, unsurprising that this study demonstrated good variability and reliability. We also measured VA across the 3 d of testing, and while this measure showed very good variability (CV: 9.1%), reliability was poor (ICC: 0.48). Allen et al. (18) reported good reliability of VA of the elbow flexors using supramaximal stimulation. In the current study, we used submaximal transcutaneous stimulation when assessing VA, rather than supramaximal transcutaneous or motor nerve stimulation (26). This approach was chosen to minimize the discomfort associated with twitch interpolation and streamline the protocol. While submaximal and maximal stimulations should provide similar estimates of VA in unfatigued muscle (31), this may not be the case (26) and may explain the poor reliability of the measure. Despite this, our measures of QMVC-D showed very good variability and excellent reliability, suggesting maximal efforts were performed by the participants on each occasion and the absolute forces used during endurance testing were correct.

While we observed strong correlations between dominant and nondominant limb strength in both the upper and lower limbs, as well as between the hand grip and quadriceps strength, we did not observe any relationships between strength and volitional or nonvolitional measures of endurance in the upper or lower limbs. While it may be expected that there is an inverse relationship between strength and endurance, the results in the literature are inconsistent. Hunter and Enoka (32) observed that stronger subjects had lower endurance in the elbow flexors and hand muscles, while West et al. (33) found no relationship between endurance and handgrip strength across a range of contraction intensities ranging from 30% to 75% of MVC. We also observed no relationship between volitional hand grip endurance and volitional quadriceps endurance. While this may have been due to the different protocols used in the current study (sustained at 50% HGMVC vs intermittent at 60% QMVC), previous studies have reported no agreement in endurance capacity between the lower and upper limb musculature (10,34). Such a lack of agreement most likely reflects variations in fiber type composition and motor unit size between muscle groups and highlights the need to use muscle group-specific measures of muscle endurance rather than relying on extrapolation.

Measurements of endurance may be affected by whether sustained or repeated maximal or submaximal contractions are employed due to differences in fiber type oxidative capacity, motor unit recruitment, and muscle blood flow (35). In the current study, we used submaximal contractions to assess muscle endurance, thereby reducing the high level of participant motivation required when producing repeated maximal contractions. This method also reduces the effects of blood flow occlusion that can occur with higher levels of contraction (35,36). We observed no relationship between volitional and nonvolitional measures of quadriceps endurance. This lack of agreement between volitional and nonvolitional measures of endurance most likely reflects the different underlying mechanisms in operation during repeated volitional and nonvolitional muscle contraction. Repeated volitional contractions involve both central and peripheral mechanisms, which influence force production (4) and determine muscle endurance. However, the electrical stimulation used during nonvolitional assessment isolates the peripheral component and removes the effects of central nervous system mechanisms and any subjective influences associated with subject motivation. Stoffels et al. (37) observed no relationship between volitional and nonvolitional measures of quadriceps endurance in patients with COPD. They attributed their findings to the greater levels of breathlessness observed in their patient cohort during volitional compared with nonvolitional testing, which may have affected the participants’ ability to undertake volitional testing successfully, and this may have contributed to the lack of relationship between the modes of endurance assessment.

We used a similar electrical stimulation protocol to that employed by Burke et al. (38) to study muscle fatigue in the cat gastrocnemius muscle (40 Hz, 330 ms on, 670 ms off). Unlike the current study, Burke et al. (38) observed significant reductions in muscle tension after 30 trains; however, it is important to acknowledge that their study was in an animal preparation and involved motor nerve stimulation. Unlike Burke et al. (38), we used 30 Hz stimulation frequency due to it being representative of motor neuron firing frequencies during strong contractions in human skeletal muscle (39). When using rTES to induce muscle fatigue, there is the potential for impaired neuromuscular junction transmission or action potential propagation failure. Such failure recovers rapidly after a contraction (40), so using an intermittent stimulation protocol with recovery intervals alongside low-frequency stimulation (30 Hz) minimizes the likelihood of such transmission failure. Although we did not specifically examine the underlying cause of force loss during the protocol, we believe that low-frequency fatigue is the most likely cause of the observed loss in tension generation.

Few studies have previously employed nonvolitional techniques to assess muscle endurance. Campbell et al. (17) compared quadriceps muscle endurance in patients with idiopathic inflammatory myopathies and age-matched controls using the same protocol of repetitive transcutaneous electrical stimulation employed in the current study. While muscle strength was lower and perceived fatigue greater in the idiopathic inflammatory myopathies patients, there was no difference in the time for quadriceps force to fall to 70% between groups (65 s in patients vs 61 s in healthy controls). Although QET70 was longer in the current study, this may reflect our younger cohort. Swallow et al. (8) compared quadriceps muscle endurance in patients with severe COPD and age-matched controls using repetitive transcutaneous magnetic stimulation delivered at 30 Hz, 2 s on, 3 s off, for 50 trains. The time for quadriceps force to fall to 70% was significantly shorter in COPD compared with healthy controls group (56 vs 121 s, respectively) and they also observed positive significant correlations between the time for quadriceps force to fall to 70% and type I fiber proportions and oxidative to glycolytic enzyme activity ratio, consistent with the changes in fiber type and metabolic profile seen in COPD (41,42). As part of a larger study examining temporal changes in paralyzed muscle fatigue following SCI, Shields et al. (43) examined the between-day reliability of a modified Burke fatigue protocol (20 HZ, 330 ms on 670 ms off for 125 s) in the soleus muscle using supramaximal electrical tibial nerve stimulation. They reported excellent reliability (0.96 ICC) between two occasions and were therefore confident that changes in muscle fatigability over time following SCI were due to physiological change rather than day-to-day variability.

Previous research has shown that females have greater muscle endurance compared with males (44), and neuromuscular function and fatigability may vary across the menstrual cycle (45). Ansdell et al. (45), using a similar fatigue protocol to the current study (intermittent quadriceps contraction, 3 s on, 2 s off, at 60% MVC), showed that time to task failure varied significantly across the menstrual cycle in young eumenorrheic females. We studied 14 healthy subjects, of whom four were female. This was a convenience sample as the study was not designed to examine sex differences in the variability and reliability of volitional or nonvolitional techniques to assess muscle endurance. The limited number of female participants did not, therefore, allow robust analysis, and as a consequence, we did not control for the phase of the menstrual cycle between testing days (46).

## CONCLUSIONS

We have shown that the variability and reliability of both volitional and nonvolitional techniques are at least fair and, in most cases, good or higher. These results suggest that research can confidently utilize these methods to investigate muscle endurance in healthy populations, with future studies warranted to determine reliability in clinical populations. Nonvolitional transcutaneous electrical stimulation potentially provides a reliable measure of muscle endurance in cohorts unable to perform volitional maneuvers.

The study was funded by Guy’s and St Thomas’ Charitable Trust. T. C. is part-funded by the National Institute for Health Research (NIHR) Biomedical Research Centre at South London and Maudsley NHS Foundation Trust, King’s College London, UK. E. M. was supported by a King’s College London Undergraduate Research Fellowship (KURF). No conflicts of interest were disclosed. G. F. R. conceived and designed research; A. J. M. and E. M. performed experiments; A. J. M. and G. F. R. analyzed data, interpreted results of experiments, prepared figures, and drafted manuscript; A. J. M., E. M., C. J. J., T. C., and G. F. R. edited and revised the manuscript and approved the final version of the manuscript. Data will be made available upon reasonable request to the corresponding author. The results of the study are presented clearly, honestly, and without fabrication, falsification, or inappropriate data manipulation. The results of the present study do not constitute endorsement by the American College of Sports Medicine.
